# Biphenotypic Acute Leukemia versus Myeloid Antigen-Positive ALL: Clinical Relevance of WHO Criteria for Mixed Phenotype Acute Leukemia

**DOI:** 10.1155/2018/7456378

**Published:** 2018-07-24

**Authors:** William A. Hammond, Pooja Advani, Rhett P. Ketterling, Daniel Van Dyke, James M. Foran, Liuyan Jiang

**Affiliations:** ^1^Division of Hematology and Oncology, Mayo Clinic, Jacksonville, FL, USA; ^2^Baptist MD Anderson Cancer Center, Jacksonville, FL, USA; ^3^Division of Laboratory Medicine and Pathology, Mayo Clinic, Rochester, MN, USA; ^4^Division of Laboratory Medicine and Pathology, Mayo Clinic, Jacksonville, FL, USA

## Abstract

Updated WHO criteria define mixed phenotype acute leukemia (MPAL) with more stringent diagnostic criteria than the formerly described entity biphenotypic acute leukemia (BAL). The changes in diagnostic criteria influence management by assigning weight to aberrantly expressed markers and minimizing expression of myeloid markers other than myeloperoxidase (MPO), potentially foregoing consolidative allogeneic transplant for an otherwise “favorable” lymphoid phenotypic leukemia. We present a case of MPO-negative, myeloid antigen-positive acute lymphoblastic leukemia who progressed with refractory phenotypic acute myeloid leukemia while receiving lymphoid-directed therapy and discuss concerns raised by the adoption of the new, more stringent diagnostic criteria for BAL.

## 1. Introduction

Acute leukemia (AL) refers to a broad category of diseases defined by the clonal, malignant proliferation of hematopoietic progenitor cells with aberrant differentiation and is categorized by the World Health Organization (WHO) as myeloid, lymphoid, or those with ambiguous lineage. Within the category of ambiguous lineage are the mixed phenotype acute leukemias (MPALs) [1]. The term “MPAL” was introduced in the WHO classification 4th edition, 2008, and includes the former clinical entities of bilineal and biphenotypic acute leukemia (BAL). Bilineal refers to two separate, concomitant blast populations with distinctly different lineages, whereas BAL refers to a single blast population with aberrant coexpression of both myeloid- and lymphoid-specific markers. The diagnosis of MPAL is unchanged in the 2017 WHO revised 4th edition [2].

The diagnostic criteria for MPAL are now more stringent and exclude some cases that would formerly have been considered as BAL. Historically, BAL comprises up to 5% of all leukemias [3]; however, when strictly defined by the current WHO criteria, the incidence of MPAL may be as low as 0.5–2.4% [4, 5]. The criteria for MPAL exclude ALs with aberrant expression of antigens of an alternate lineage, which can be observed in as many as one-third of all B-lineage ALL cases when a limited myeloid immunophenotypic panel is applied, including only CD13 and CD33, known as myeloid antigen-positive (My+) ALL [6]. Leukemic blasts may also betray lineage fidelity, often in a consistent or predictable manner, leading to the distinction within the current WHO classification of MPAL with *BCR-ABL1* and with *MLL* (i.e., t[v;11q23]) rearrangements as unique entities. At any point during the course of AL, a new blast population may arise with an antigen-expression profile characteristic of a different lineage, referred to as lineage switch. To illustrate potential pitfalls and challenges of the current revised classification, we report a case of lineage switch from My+ ALL to an AML phenotype during intensive ALL-directed chemotherapy, with persistence of an underlying *CDKN2A* deletion.

## 2. Case

A 23-year-old man presented in 2014 with a white blood cell count of 34 × 10^9^/L with 87% circulating blasts by manual differential count. Flow cytometric analysis on the peripheral blood (PB) revealed 89.6% blasts by CD45/SSC gating. The blasts expressed CD10, CD19, CD34, HLA-DR, and CD20 (dim); partially expressed CD13, CD15, and CD33; and did not express CD2, CD7, CD56, and CD117. A subsequent bone marrow (BM) biopsy was done the next day but was a dry tap; therefore, flow cytometry and other cytogenetic studies were not performed on the BM sample. Immunohistochemistry (IHC) studies were performed on the BM biopsy showing 95% blasts positive for CD79a, PAX-5, and TdT, and negative for CD20 and myeloperoxidase (MPO). The morphology and immunophenotype of the blasts in the peripheral blood and bone marrow biopsy were consistent with B-lineage lymphoblastic leukemia. We did perform FISH analysis with a B-ALL panel on the peripheral blood specimen which revealed *CDKN2A* (p16 at 9q21) gene deletion on one or both chromosomes 9. Fusion of *BCR* and *ABL1* was not detected. Intensive chemotherapy was initiated according to the CALGB 10403 “Adolescent Young Adult” regimen [7], and the patient achieved complete remission. He then proceeded to consolidation without consideration of allogeneic hematopoietic cell transplantation (allo-HCT) based on standard of care for B-cell ALL with favorable cytogenetic and molecular profile.

**Figure 1:**
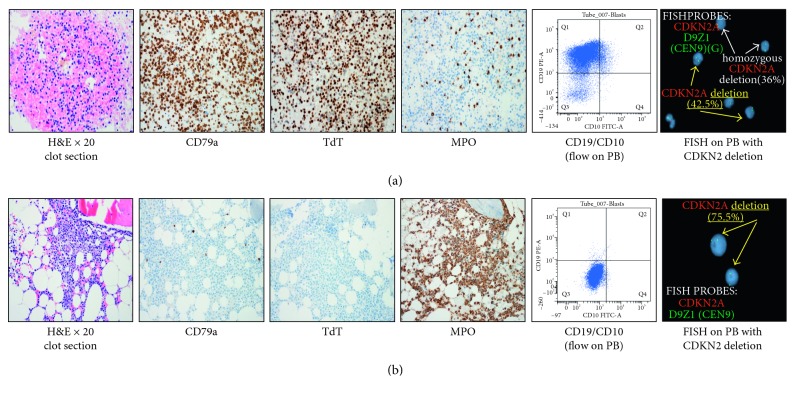
Histology and immunohistochemistry (IHC) studies on bone marrow biopsies. Flow cytometry analysis and FISH studies on peripheral blood specimens from (a) diagnosis and (b) relapse at 24 weeks. (a) This series shows the phenotype of B-cell ALL, staining positive for CD79a and TdT but negative for MPO. Flow cytometry shows a blast population that is strongly CD19 positive and weakly CD10 positive. The FISH study shows two blast populations: a heterozygous deletion in 42.5% of nuclei [9p-(CDKN2Ax1,D9Z1x2)] and a homozygous deletion in 36% of nuclei [9p-x2(CDKN2Ax0,D9Z1x2)]. (b) This series demonstrates a myeloid leukemia phenotype with strong MPO staining and lack of CD19 and CD10 expression by flow. The FISH shows persistence of a heterozygous CDKN2A gene deletion [9p-(CDKN2Ax1,D9Z1x2)] present in 75.5% of nuclei tested. ALL, acute lymphoblastic leukemia; CDKN2A, cyclin-dependent kinase inhibitor 2A; FISH, fluorescence in situ hybridization; TdT, terminal deoxynucleotidyl transferase; MPO, myeloperoxidase.

However, the patient had prolonged cytopenias during consolidation therapy culminating in treatment delay. In early January of 2015 (approximately 24 weeks after initial diagnosis), the patient's complete blood count revealed 27% blasts. Flow cytometry analysis was performed on the PB; the blasts expressed HLA-DR, CD15, CD33, and CD117; partially expressed CD13 and CD56; and did not express CD2, CD3, CD5, CD7, CD10, CD19, CD20, or CD34. A restaging BM biopsy was performed the same day; unfortunately, it was again a dry tap, so flow cytometric analysis could not be performed. The histology examination of the BM biopsy revealed a hypocellular (30%) marrow with 85% recurrent leukemic blasts. IHC showed the blasts were now positive for MPO, while CD10, PAX-5 CD20, CD79a, and TdT were negative. The overall findings on PB and BM biopsy were consistent with a lineage switch to acute myeloid leukemia (AML) (Figure 1). Although it was a dry tap, a very small amount of aspiration specimen was obtained in the EDTA tube. Despite few marrow spicules on the aspiration smear, cytogenetic analysis and FISH studies were performed. FISH analysis with both B-ALL and AML panels showed persistence of a heterozygous *CDKN2A* deletion, plus the acquisition of a *TP53* deletion, and 7q and 17q duplications. Cytogenetic studies now showed a complex karyotype in 18/20 metaphases, 47,X,-Y,add(1)(p36.1),+18,+add(18)(q23) [12]/47–48,X,−Y,del(1)(p32p36.1),add(11)(p11.2),+18,+18[cp6]/46,XY [2].

Intensive salvage chemotherapy was initiated with the MEC regimen [8], and nadir BM evaluation was hypocellular without blasts, although FISH demonstrated low-level *CDKN2A* deletion, consistent with minimal residual disease. Ten days later, a rising PB blast percentage prompted another BM biopsy which showed persistence of AL with the same phenotype and FISH with *CDKN2A* deletion. Further intensive chemotherapy was initiated, but the patient ultimately died of respiratory failure and refractory AL.

## 3. Discussion

AL frequently presents with aberrant expression of antigens despite putative lineage fidelity. My+ ALL describes a heterogeneous group with aberrant expression of a small number of cross-lineage markers (<2 points) by earliest definition from the European Group for the Immunological Characterization of Leukemias (EGIL) [9] and later adopted by the WHO in the 2001 guidelines [10]. The impact of aberrant antigen expression short of BAL has been investigated and does not appear to alter therapy or prognosis but may be useful for monitoring of minimal residual disease [11].

**Figure 2:**
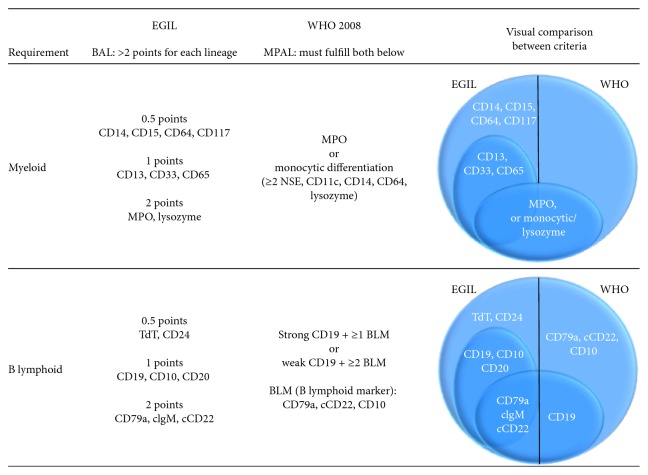
Comparison of EGIL criteria for BAL and the 2008 WHO criteria for MPAL. BAL, biphenotypic acute leukemia; EGIL, European Group for the Immunological Characterization of Leukemias; MPAL, mixed phenotype acute leukemia.

We present a case of AL that formerly would have been considered BAL by the EGIL criteria but was diagnosed as My+ ALL using current WHO criteria, not meeting the criteria for MPAL. The initial blast population expressed sufficient lymphoid (CD79a, CD19, CD10, and TdT) and myeloid (CD13, CD33, and CD15) antigens to qualify as biphenotypic under former terminology; however, in the absence of MPO, the only marker currently considered definitive for myeloid categorization, it could not be considered MPAL. At the time of disease relapse after ALL-directed therapy, a clear lineage switch to an AML phenotype had occurred. This subsequent recurrence suggests that an initial diagnosis of BAL as recognized by the EGIL criteria may have been more clinically applicable for this patient.  Figure 2 compares the former and current diagnostic requirements of BAL and MPAL under the EGIL and WHO criteria, respectively, and highlights the stringency of the modern WHO criteria for MPAL.

This case further illustrates an example of lineage switch during active therapy for AL and not secondary AML, which was confirmed by persistence of CDKN2A deletion in both leukemias. The mechanism of phenotypic evolution remains unknown, but the emergence of a second preexisting leukemic population selected by treatment of the primary disease or clonal selection could account for the evolution of this disease. Another theory suggests induced changes within the progenitor cell that may be therapy independent. Lineage switches have been documented in 6–9% of AL cases at relapse, and the time from initial treatment to relapse has been demonstrated to correlate with a lack of response to subsequent treatment, short duration of second remission, and short event-free survival [12].

Deletion at 9p21 is relatively frequent in adult ALL and is observed in 10–15% of cases. This deletion affects the tumor suppressor gene *CDKN2A*, which encodes for p16^INK4a^ and p14^ARF^ and results in abnormal regulation of the cell cycle by preventing the phosphorylation of the retinoblastoma protein. The prognostic significance of del(9p21) in the large prospective UKALLXII/E2993 trial [13] is associated with superior 5-year overall survival (OS). Conversely, del(9p21) is uncommon in AML, occurs in only 2–5% of cases [14], and portends a poor prognosis with shorter duration of complete response and lower event-free OS [15].

BALs have a worse prognosis and historically have been treated with either myeloid or lymphoid therapy with early consideration of allo-HCT [3, 4]. This patient received lymphoid-directed induction therapy with an intensive AYA regimen, since the *CDKN2A* deletion was not clearly an adverse marker. In retrospect, the emergence of a resistant myeloid clone during ALL-based therapy suggests the designation of BAL may have been appropriate and could have guided therapy, including consideration of early allo-HCT.

This case challenges the current definition of MPAL, which may be too restrictive clinically, and the classification of cases that formerly met the criteria for BAL. The current WHO criteria may not consistently identify all MPAL patients, limiting the clinical utility of this AL designation.
